# Burden and Determinants of Urinary Incontinence and Its Quality‐of‐Life Impact Among Women in Saudi Arabia: A Cross‐Sectional Online Analysis

**DOI:** 10.1155/ogi/3195077

**Published:** 2026-09-30

**Authors:** Elhadi Miskeen, Atheer Hameed Alqarni, Dalia Alqarni, Abdullah Ayed, Reema Dakhilallah Alghanmi, Raneem Raja Alsaeedi, Ruwayda Hamad Alharbi, Daniah Ahmed Aljehani, Amal Fayez Hamuman, Ghadah F. Alshalan, Deema Munif Alotaibi, Layan A. Alshahrani, Raed Ibrahim Alwadai, Ghada Ayed Mohammed Alqarni, Omar Safar

**Affiliations:** ^1^ Department of Obstetrics and Gynecology College of Medicine University of Bisha Bisha Saudi Arabia ub.edu.sa; ^2^ Department of Surgery College of Medicine University of Bisha Bisha 61922, P.O. Box 551 Saudi Arabia ub.edu.sa; ^3^ College of Medicine and Surgery Batterjee Medical College Jeddah Saudi Arabia bmc.edu.sa; ^4^ Faculty of Medicine King Abdulaziz University Jeddah Saudi Arabia kau.edu.sa; ^5^ College of Medicine Qassim University Buraidah Saudi Arabia qu.edu.sa; ^6^ College of Medicine King Saud University Riyadh 11362 Saudi Arabia ksu.edu.sa; ^7^ College of Medicine Princess Nourah bint Abdulrahman University Riyadh Saudi Arabia pnu.edu.sa; ^8^ Urology Department King Abdullah Hospital Bisha Asir Saudi Arabia; ^9^ Urology Department Armed Forces Hospital Southern Region Khamis Mushayt Saudi Arabia afhsr.med.sa

**Keywords:** prevalence, risk factors, urinary incontinence, women’s health

## Abstract

**Background:**

Urinary incontinence (UI) is a prevalent yet underreported condition significantly impacting women’s quality of life. In Saudi Arabia, cultural stigma often prevents help‐seeking, and comprehensive data on its impact are limited. This study aimed to estimate the prevalence and severity of UI among women in Saudi Arabia, identify associated medical and obstetric risk factors, and evaluate its impact on quality of life in order to inform a policy and service framework for integrated, stigma‐sensitive care.

**Methods:**

A cross‐sectional study was conducted among 629 adult women across Saudi Arabia using an online, self‐administered questionnaire. The instrument included sociodemographic sections, the validated International Consultation on Incontinence Questionnaire‐Short Form (ICIQ‐UI SF), and assessments of risk factors and quality‐of‐life impact. Data were analyzed using descriptive statistics, chi‐square tests, and multiple logistic regression.

**Results:**

Regarding the amount leaked among the 291 women who reported incontinence, most reported small‐volume leakage: 238 (81.8%), while moderate 51 (17.5%) and large amounts 2 (0.7%) were relatively rare. Key risk factors identified included higher age, overweight/obesity (with 60.7% of frequent UI cases being overweight), and multiparity (46.3% of all women had ≥ 3 deliveries). The impact on quality of life was substantial: 25.4% reported an impact on their social life, and 21.0% reported feelings of anxiety or nervousness. Multivariable analysis revealed that severe social impact was independently associated with high leakage frequency (AOR = 4.25, *p* < 0.001), higher perceived interference (AOR = 1.28, *p* < 0.001), and experiencing anxiety (AOR = 3.02, *p* = 0.005).

**Conclusion:**

UI is highly prevalent among women in Saudi Arabia, with significant physical and psychosocial consequences. Symptom severity and psychological distress are the primary drivers of social impairment. There is an urgent need for targeted public health initiatives to destigmatize UI, raise awareness, and improve access to treatment. These findings support a national framework that embeds routine UI screening in primary care, strengthens access to pelvic‐floor and weight‐management services, and integrates mental‐health support and culturally sensitive awareness campaigns to reduce stigma and improve quality of life.

## 1. Introduction

Urinary incontinence (UI) is described as the involuntary passage of urine, usually at an inappropriate time and place [1]. There are five main subtypes of UI, categorized by accompanying symptoms or the cause of the involuntary leakage. The subtypes of UI include stress UI (SUI), which is associated with physical pressure, urge UI (UUI), which is preceded by a strong and sudden urge to urinate, and mixed UI (MUI), characterized by both SUI and UUI [2]. In addition, there are two other categories, including overflow UI, which involves frequent dribbling from a bladder that doesn’t empty, and functional UI, which occurs when the patient cannot reach a toilet in time due to physical or mental issues [3]. Regardless of the type of UI that affects a person, it can be an embarrassing situation that can significantly affect someone’s daily life. UI is a prevalent health concern among women globally, though reported prevalence rates vary widely from approximately 5% to 70% due to differences in definitions, study populations, and sampling procedures. Most population‐based studies report a prevalence of any UI in the range of 25%–45%, with figures strongly correlated to age; over 40% of women aged ≥ 70 are affected, and rates are even higher among the elderly in nursing homes [4]. Given the increase in mean life expectancy, the overall prevalence of UI in women is expected to rise in the future. Within this global context, recent studies in Saudi Arabia indicate a substantial burden among women of reproductive age, with reported prevalence rates ranging from approximately 31% to 32% in both general and pregnant populations [5, 6]. Given the increase in mean life expectancy, the overall prevalence of UI in women is expected to rise in the future. A systematic review of studies from the Gulf region similarly reported a high prevalence of UI among women, ranging from 20.3% to 54.5%, underscoring a consistent regional challenge [7].

Existing research indicates that UI is about two to three times more common in women [8]. Although the condition becomes more common with age, it can also affect women of any age. Other risk factors include being a smoker, being overweight, and a family history of the condition [9]. Due to its nature, the condition can cause significant physical discomfort and psychological distress. Studies have associated UI with various psychological issues like social isolation, depression, and anxiety, and Porto et al. specifically report that it can negatively affect sexual relationships by creating emotional barriers that limit desire and satisfaction [10]. Consequently, the condition can significantly and negatively affect a person’s quality of life (QoL) because it imposes substantial social and economic burdens. According to Reynold et al., close to $20 billion was spent on UI treatment for women in 1 year in the United States alone [11]. This is an illustration of the major health challenge posed by UI. In Saudi Arabia, UI is significantly underreported, with most of the affected women choosing not to seek medical advice. Some factors cited as responsible for the high level of underreporting include embarrassment, social stigma, and cultural and religious demands for cleanliness [5]. Despite previous research on the prevalence and risk factors of UI in Saudi women, there is a significant gap in the literature regarding its impact on the QoL, psychological well‐being, and healthcare‐seeking behaviors. A deeper understanding of the sociodemographic associations, symptom severity and frequency, psychological consequences, and barriers to care is essential to inform healthcare policies and develop targeted interventions. This study aimed to estimate the prevalence and severity of UI among women in Saudi Arabia, identify associated medical and obstetric risk factors, and evaluate its impact on the QoL in order to inform a policy and service framework for integrated, stigma‐sensitive care.

## 2. Methodology

### 2.1. Study Design

An extensive, cross‐sectional study was perfectly performed in compliance with the STROBE statement (appendix). The analytical study was rigorously carried out for several months (May to September; 2025), with the main focus on calculating the proportions of UI prevalence, scope, and recognition of adverse number of associated risk factors, and thoroughly assessing consequent ill effects (manifested as reduced QoL) among women inhabiting these culturally dynamic parts in an ethnically diversified country like Saudi Arabia. The research study deliberately included women all over the five main geographical regions of the country—the Central, Eastern, Western, Northern, and Southern regions—and was designed to contribute to significantly increasing the geographical representativeness of the findings and to be able to capture regional heterogeneity in sociodemographic characteristics and health conditions that are likely to affect the presentation of UI.

### 2.2. Sampling and Sample Size

The respondents in this research were adult females aged 18 years or older, with residence status in Saudi Arabia during the completion of the questionnaires. To reduce potential selection bias and maximize the overall representativeness of the sample, an adaptive sampling approach was used utilizing a combination of online convenience sampling methods in conjunction with quota‐based stratification that was weighted by geographic region and cut across broad age categories, i.e., 18–25 years, 26–35 years, 36–45 years, 46–55 years, and those > 55. It also employed monitoring of the recruitment to ensure that minimum numbers of participants were achieved for each stratum (to enhance equitable sampling across demographics). The sample size was carefully calculated using a single‐proportion formula, taking the expected prevalence of UI as 50% to ensure that the study would have an adequate sample size. This was based on a 95% confidence level (*Z* = 1.96) and a 5% margin of error, requiring at least 385 women in order to ensure that the statistical analysis is valid. Finally, the analytic sample was composed of 629 respondents in all, resulting in a considerable improvement of the accuracy of the estimates obtained and increasing statistical power for multivariable analysis.

Figure 1 presents a flowchart summarizing the participant recruitment process. A total of 629 eligible women completed the full questionnaire and were included in the final analytic sample. Quota‐based stratification by geographic region and age category was monitored throughout the recruitment period to ensure adequate representation across all predefined strata.

**FIGURE 1:**
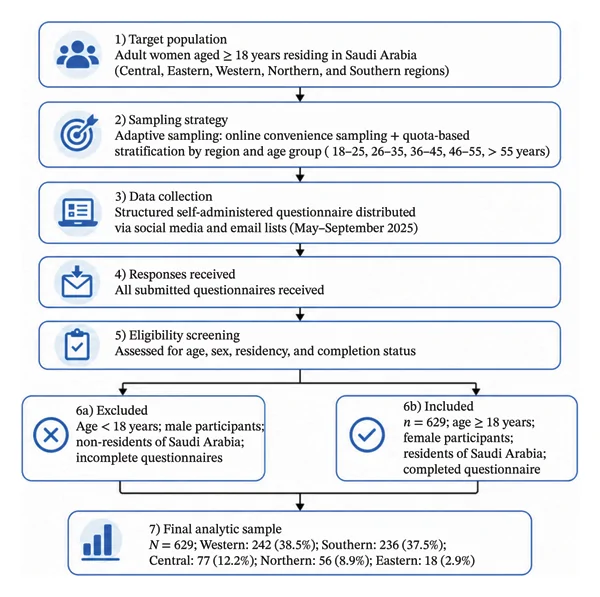
Participant recruitment process.

### 2.3. Questionnaire Development and Validation

This study employed a questionnaire created by obstetricians/gynecologists, urologists, and medical students. It started with a literature review to identify the appropriate range of considerations and aspects of risk factors associated with UI, followed by an expert consensus for item selection and cultural acceptability by the Saudi population. For the UI‐specific part, we included the Arabic version of the validated International Consultation on Incontinence Questionnaire‐Urinary Incontinence Short Form (ICIQ‐UI SF), which has demonstrated well‐established psychometric performance among Saudi subjects (Cronbach’s alpha = 0.83) [12]. The other sections (sociodemographic characteristics, obstetric history, and impact in specific domains) were constructed for this study with reference to the literature and clinical experience.

To assess face validity and clarity, the complete questionnaire was piloted with 20 participants representative of the target population. Participants provided feedback on comprehension, relevance, and cultural appropriateness of all items. The pilot testing indicated that the questionnaire was clear and understandable, with no questions requiring modification. The average completion time was 5–6 min, indicating acceptable respondent burden. Content validity was established through expert review by the multidisciplinary research team, ensuring comprehensive coverage of all relevant domains of UI impact, including physical, social, psychological, and functional aspects.

### 2.4. Operational Definitions and Measurements

UI was identified as any reported involuntary loss of urine according to the International Continence Society definition. For analytical purposes, participants were classified as experiencing “frequent UI” if they reported leaking on the ICIQ‐UI SF frequency item “2 or 3 times a week” or more often and as experiencing “infrequent/none” if they leaked “about once a week or less often” or reported having never leaked. Body mass index (BMI) was derived from self‐reported weight and height (kg/m^2^), and further classified as underweight (< 18.5), normal (18.5–24.9), overweight (25.0–29.9), and obese (≥ 30.0) according to the WHO definition standards of BMI categories for elderly women (16).

Impact of the condition (housework, physical effort, social life, mental status, and sleep) was collected on a Likert scale from no impact to very severe and kept as a nominal variable for the outcome “severe” vs. “nonsevere” to be entered into the regression models, whereas satisfaction with care (0–10) was included as continuous in multivariable analyses.

For analysis, participants were categorized by the frequency of their UI symptoms. “Any UI” was defined as any reported involuntary loss of urine. “Frequent UI” was defined as leakage occurring “2 or 3 times a week” or more often; participants with leakage “about once a week or less often” were categorized as having infrequent or no UI for the purposes of the frequent UI analysis.

*Any UI (Overall Prevalence)*: Defined as any reported involuntary loss of urine, regardless of frequency, severity, or amount. This includes all participants who answered positively to experiencing urine leakage on the ICIQ‐UI SF, ranging from “about once a week or less” to “all the time.”

*Frequent UI (Clinically Significant Prevalence)*: Defined as leakage occurring “2 or 3 times a week” or more often (i.e., ≥ 2–3 times weekly). This category represents a subgroup of the “Any UI” group and reflects a clinically significant burden requiring active intervention. Participants who reported leakage “about once a week or less often” were categorized as having infrequent UI for analytical purposes.

### 2.5. Data Analysis

SPSS 29 was used for statistical analysis. Summary statistics were calculated for baseline demographics, UI prevalence, and domain‐specific impact; continuous measures were described as mean ± SD or median (interquartile range IQR) if distributed normally, whereas frequencies and percentages (%) were reported for categorical variables. Associations for potential risk factors and UI (and) frequency as dichotomous variables at bivariable analysis were explored using Pearson’s chi‐square test or Fisher’s exact test for categorical examined variables and the independent‐samples *t* test or Mann–Whitney *U* test for continuous ones. Factors associated with UI or having a significant social impact (*p* < 0.20 in bivariable analyses, clinically meaningful variables such as age, BMI, parity, menopause, and chronic illnesses) were included in multivariable logistic models to determine the independent predictors; absence of collinearity was tested using the variance inflation factor, and model calibration was assessed by the Hosmer–Lemeshow test. Results are presented as adjusted odds ratios (AOR) with 95% confidence intervals (CI), and a two‐tailed *p* value < 0.05 was considered statistically significant.

*Declaration of AI-assisted tools*: During the preparation of this study, the authors used Grammarly for grammar checking, spelling correction, and proofreading. This tool was used solely for language enhancement and did not contribute to the generation of intellectual content, data analysis, or scientific interpretation. The authors completely revised the manuscript, and they are the only responsible persons for the final one.

## 3. Results

The results address the study’s goal of assessing the prevalence of UI, its associated risk factors, and its impact on women’s daily lives in Saudi Arabia. The sociodemographic characteristics of the 629 participants are described to contextualize the sample, followed by detailed findings on the prevalence, frequency, and volume of urinary leakage and patterns of incontinence. Associations between UI and key medical, obstetric, and lifestyle factors are then examined, and the consequences for daily activities, social functioning, and psychological well‐being are presented, including multivariable analyses of determinants of severe social impairment.

The overall prevalence of any UI (defined as any reported involuntary urine loss) was 46.3% (291/629). Within this group, frequent UI (defined as leakage occurring ≥ 2–3 times per week or more often) was reported by 27.5% (173/629) of all participants, representing a clinically significant subgroup.

The following is the overall prevalence of UI among the study participants. More than half of the respondents (53.74%) reported not experiencing UI, while 46.26% indicated symptoms of urine leakage, showing that it affects a substantial proportion of individuals.

Table 1 summarizes the sociodemographic characteristics of the 629 women included in the study. The majority were Saudi nationals (581, 92.4%), whereas 48 (7.6%) were non‐Saudi. Most participants belonged to the 18–45 years age group (419, 66.6%), while 153 (24.3%) were in the 46–54 years group, and 57 (9.1%) were in the 55 years or older group. In terms of BMI, 227 (36.1%) had normal weight, 304 (48.3%) were overweight, and 59 (9.4%) were classified as obese. Similarly, the largest proportions of participants lived in the Western (242, 38.5%) and Southern (236, 37.5%) regions. Moreover, more than half were married (376, 59.7%), while 218 (34.7%) were single. Most women held a university degree (490, 77.9%), with an additional 92 (14.6%) having completed secondary education. Regarding occupation, 228 (36.2%) were employed, 148 (23.6%) were students, 188 (29.9%) were unemployed, and 65 (10.3%) were retired.

**TABLE 1:** Sociodemographic characteristics of study participants (*N* = 629).

Variables	Categories	*n* (%)
Nationality	Saudi	581 (92.4%)
	Non‐Saudi	48 (7.6%)

Age	18–25 years	191 (30.4%)
	26–35 years	102 (16.2%)
	36–45 years	126 (20.0%)
	46–55 years	153 (24.3%)
	Above 55 years	57 (9.1%)

BMI Category	Underweight	39 (6.2%)
	Normal	227 (36.1%)
	Overweight	304 (48.3%)
	Obese	59 (9.4%)

Place of residence	Central Region	77 (12.2%)
	Eastern Region	18 (2.9%)
	Northern Region	56 (8.9%)
	Southern Region	236 (37.5%)
	Western Region	242 (38.5%)

Marital Status	Divorced	13 (2.1%)
	Married	376 (59.7%)
	Single	218 (34.7%)
	Widow	22 (3.5%)

Level of Education	Uneducated	6 (1.0%)
	Primary	16 (2.5%)
	Secondary school	25 (4.0%)
	High school	92 (14.6%)
	University	490 (77.9%)

Occupation	Employee	228 (36.2%)
	Unemployed	188 (29.9%)
	Retired	65 (10.3%)
	Student	148 (23.6%)

*Note:* Data have been presented has *n* and %.

Table 2: Over half of the participants (338, 53.7%) reported never experiencing urinary leakage, while 118 (18.8%) reported leakage about once a week or less. More frequent episodes were less common, with 55 (8.7%) leaking two to three times per week, 37 (5.9%) once daily, 64 (10.2%) several times daily, and 17 (2.7%) experiencing leakage continuously. Regarding the amount leaked among the 291 participants with UI, the majority reported small‐volume leakage (238, 81.8%), while moderate (51, 17.5%) and large amounts (2, 0.7%) were relatively rare. When assessing the impact of UI on daily life using a 0–10 scale, more than half (340, 54.1%) reported no interference at all (score 0). Scores indicating mild impact (1–3) were reported by 101 (21.2%) participants, whereas higher interference levels (scores 4–10) were less frequent, suggesting that UI had a minimal effect on daily functioning for the majority. Regarding situations that trigger leakage, 321 (51.0%) reported no leakage at any time. Among those who did, the most common triggers included coughing or sneezing (116, 18.4%) and physical activity or exercise (107, 17.0%), followed by urgency before reaching the toilet (85, 13.5%) and leakage during sleep (79, 12.6%). Less common scenarios included leakage after urination 28 (4.5%), for no clear reason 38 (6.0%), or continuously 47 (7.5%).

**TABLE 2:** Prevalence and patterns of urinary incontinence among participants (*N* = 629).

UI assessment variables	UI	(%)
How often do you leak urine?	Never	338 (53.7%)
	About once a week or less often	118 (18.8%)
	2 or 3 times a week	55 (8.7%)
	About once a day	37 (5.9%)
	Several times a day	64 (10.2%)
	All the time	17 (2.7%)

How much urine do you usually leak? (*N* = 291)	A small amount	238 (81.8%)
	A moderate amount	51 (17.5%)
	A large amount	2 (0.7%)

Overall, how much does leaking urine interfere with your everyday life? (Scale from 0 to 10, where 0 = not at all, 10 = a great deal)	0	340 (54.1%)
	1	61 (9.7%)
	2	35 (5.6%)
	3	37 (5.9%)
	4	29 (4.6%)
	5	38 (6.0%)
	6	28 (4.5%)
	7	24 (3.8%)
	8	12 (1.9%)
	9	5 (0.8%)
	10	20 (3.2%)

When does urine leak? (select all that apply)	Never: Urine does not leak	321 (51.0%)
	Before you can get to the toilet	85 (13.5%)
	When you cough or sneeze	116 (18.4%)
	When you are asleep	79 (12.6%)
	When you are physically active/exercising	107 (17.0%)
	When you have finished urinating and are dressed	28 (4.5%)
	For no obvious reason	38 (6.0%)
	All the time	47 (7.5%)

*Note:* Data ha been presented as count (*n*) and frequency (%).

Figure 2 shows that UI had a measurable effect on several aspects of daily life and psychological well‐being, although most women still reported no impact in each individual domain. The most affected areas were physical activity, where 27.7% reported some degree of limitation; social life, where 25.4% reported mild‐to‐severe impact; and sleep, which was altered in 24.8% of participants. Psychological distress was also notable, with 21.0% of women reporting worry or anxiety, 8.9% feeling self‐conscious, and 7.2% reporting depressive feelings related to UI. In contrast, household duties were less affected than other domains, with only 18.4% reporting any impact and severe limitation reported by very few participants.

**FIGURE 2:**
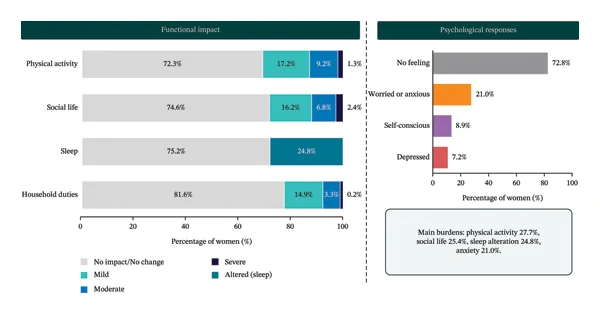
Functional and psychological impact of urinary incontinence among women in Saudi Arabia (*N* = 629).

Table 3 shows several statistically significant associations between potential risk factors and UI. Participants with chronic diseases, particularly hypertension and diabetes, had higher UI prevalence (*p* = 0.02). Obstetric factors were also strongly linked, as women with two or more deliveries, vaginal births, or cesarean sections had higher rates of UI compared to those without childbirth experience (*p* = 0.001). History of miscarriage and earlier age at first delivery were associated with increased UI risk (*p* = 0.001). Menopause was more common among participants with UI (23.7% vs. 13.0%). Chronic constipation and chronic cough were significantly associated with UI (*p* = 0.001), while smoking status showed no significant relationship (*p* = 0.626). Family history showed a strong association: 34% of those with an affected family member experienced UI, compared with 15.4% without (*p* = 0.001).

**TABLE 3:** Association between risk factors and the prevalence of urinary incontinence among participants.

Risk factors assessment variables	Risk factors patterns	Urinary incontinence		*p* value
		No	Yes	
Do you have any chronic diseases: (select all that apply)	Hypertension	15 (4.4%)	30 (10.3%)	0.020^∗^
	Diabetes	17 (5.0%)	45 (15.5%)	
	Others. (asthma [2 cases], arthritis [3 cases], hypothyroidism [15 cases], heart disease [2 cases], sickle cell anemia [2 cases], sclerosis [1 case], irritable bowel syndrome [1 case], ovarian cysts [1 case], cancer [1 case])	16 (4.7%)	12 (4.1%)	
	I don’t suffer from any chronic disease	287 (84.9%)	204 (70.1%)	

Number of deliveries:	0	177 (52.4%)	59 (20.3%)	0.001^∗^
	1	19 (5.6%)	23 (7.9%)	
	2	21 (6.2%)	41 (14.1%)	
	≥ 3	121 (35.8%)	168 (57.7%)	

Vaginal delivery	No	43 (12.7%)	38 (13.1%)	0.001^∗^
	Yes	122 (36.1%)	196 (67.4%)	
	I haven’t given birth before	173 (51.2%)	57 (19.6%)	

C‐section delivery	No	106 (31.4%)	151 (51.9%)	0.001^∗^
	Yes	62 (18.3%)	82 (28.2%)	
	I haven’t given birth before	170 (50.3%)	58 (19.9%)	

History of miscarriage:	No	84 (24.9%)	126 (43.3%)	0.001^∗^
	Yes	85 (25.1%)	108 (37.1%)	
	I haven’t given birth before	169 (50.0%)	57 (19.6%)	

Age at first delivery (years):	≤ 20	26 (7.7%)	56 (19.2%)	0.001^∗^
	21–25	94 (27.8%)	123 (42.3%)	
	≥ 26	40 (11.8%)	53 (18.2%)	
	I haven’t given birth before	178 (52.7%)	59 (20.3%)	

Have you reached menopause?	No	294 (87.0%)	222 (76.3%)	0.001^∗^
	Yes	44 (13.0%)	69 (23.7%)	

Smoking status:	No	320 (94.7%)	276 (94.8%)	0.626
	Yes	9 (2.7%)	10 (3.4%)	
	Quitted smoking	9 (2.7%)	5 (1.7%)	

History of chronic constipation:	No	289 (85.5%)	208 (71.5%)	0.001^∗^
	Yes	49 (14.5%)	83 (28.5%)	

Chronic cough:	No	324 (95.9%)	260 (89.3%)	0.001^∗^
	Yes	14 (4.1%)	31 (10.7%)	

History of any pelvic surgeries:	No	303 (89.6%)	252 (86.6%)	0.001^∗^
	Yes	35 (10.4%)	39 (13.4%)	

Family history of urinary Incontinence:	No	286 (84.6%)	192 (66.0%)	0.001^∗^
	Yes	52 (15.4%)	99 (34.0%)	

*Note:* The Chi‐square test was used to assess statistical significance. A *p* value of < 0.05 was considered statistically significant, and statistically significant values were marked with an asterisk (^∗^) in the tables.

Figure 3 shows that 116 females reported that their ability to perform household duties was affected by UI; 94 (14.9%) reported mild effects, 21 (3.3%) reported moderate effects, and 1 (0.2%) reported severe effects. In addition, 513 (81.6%) had no impact. In terms of physical activity, 455 (72.3%) reported no effect, 108 (17.2%) reported mild effects, 58 (9.2%) reported moderate effects, and 8 (1.3%) reported severe effects. In 102 cases (16.2%), social life was mildly affected; in 43 cases (6.8%), moderately affected; in 15 cases (2.4%), severely affected; and in 469 cases (74.6%), there was no influence. Of those who reported mental health, 132 (21.0%) reported feeling worried or anxious, 56 (8.9%) felt self‐conscious, and 45 (7.2%) felt depressed; 458 (72.8%) reported having no feeling. UI caused alterations in sleep patterns among 156 (24.8%) participants, while 473 (75.2%) reported no changes.

**FIGURE 3:**
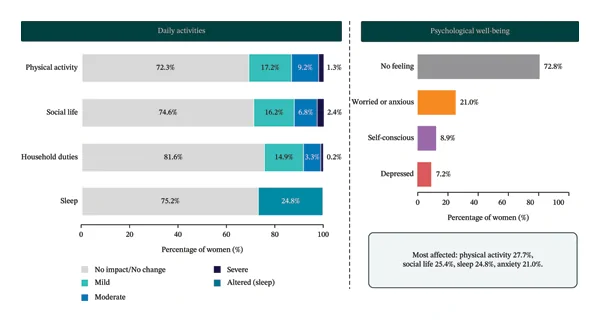
Impact of urinary incontinence on daily activities and well‐being among women in Saudi Arabia (*N* = 629).

Table 4 shows that the frequency of UI among Saudi women was significantly associated with several sociodemographic factors. Age had a strong correlation (*p* = 0.001), with higher rates among women aged 46–55 years (51, 29.5%) and 36–45 years (44, 25.4%), while younger women (18–25 years) showed the lowest prevalence (9.8%). BMI was also significantly related (*p* = 0.001); overweight 105 (60.7%) and obese 31 (17.9%) women had the highest rates. Place of residence demonstrated a significant difference (*p* = 0.004), with most cases from the Western (76, 43.9%) and Southern (45, 26.0%) regions. Marital status was significantly associated (*p* = 0.001), as married women 136 (78.6%) reported more cases than single or widowed participants. Education level also showed a significant association (*p* = 0.001), with lower education levels associated with higher prevalence. Occupation was significant (*p* = 0.001); unemployed (85, 46.4%) had the highest rate, while students (8, 4.6%) had the lowest.

**TABLE 4:** Association of the social demographic characteristics and the frequency of urinary incontinence among females in Saudi Arabia.

Variables	Categories	Frequent *n* (%)	Infrequent *n* (%)	*p* value
Nationality	Saudi	161 (93.1%)	420 (92.1%)	0.415
	Non‐Saudi	12 (6.9%)	36 (7.9%)	

Age	18–25 years	17 (9.8%)	174 (38.2%)	0.001^∗^
	26–35 years	31 (17.9%)	71 (15.6%)	
	36–45 years	44 (25.4%)	82 (18.0%)	
	46–55 years	51 (29.5%)	102 (22.4%)	
	Above 55 years	30 (17.3%)	27 (5.9%)	

BMI category	Underweight	6 (3.5%)	33 (7.2%)	0.001^∗^
	Normal	31 (17.9%)	196 (43.0%)	
	Overweight	105 (60.7%)	199 (43.6%)	
	Obese	31 (17.9%)	28 (6.1%)	

Place of residence	Central Region	27 (15.6%)	50 (11.0%)	0.004^∗^
	Eastern Region	8 (4.6%)	10 (2.2%)	
	Northern Region	17 (9.8%)	39 (8.6%)	
	Southern Region	45 (26.0%)	191 (41.9%)	
	Western Region	76 (43.9%)	166 (36.4%)	

Marital status	Divorced	3 (1.7%)	10 (2.2%)	0.001^∗^
	Married	136 (78.6%)	240 (52.6%)	
	Single	25 (14.5%)	193 (42.3%)	
	Widow	9 (5.2%)	13 (2.9%)	

Level of education	Uneducated	2 (1.2%)	4 (0.9%)	0.001^∗^
	Primary	12 (6.9%)	4 (0.9%)	
	Secondary School	20 (11.6%)	5 (1.1%)	
	High School	34 (19.7%)	58 (12.7%)	
	University and above	105 (60.7%)	385 (84.4%)	

Occupation	Employee	66 (38.2%)	162 (35.5%)	0.001^∗^
	Unemployed	85 (46.4%)	103 (22.6%)	
	Retired	14 (8.1%)	51 (11.2%)	
	Student	8 (4.6%)	140 (30.7%)	

*Note:* Data have been presented as *n* and %. Both fisher’s and Pearson Chi‐square tests were used. A *p* value of < 0.05 was considered statistically significant, and statistically significant values were marked with an asterisk (^∗^) in the tables.

Table 5 shows a multiple logistic regression analysis, which identified that the severe social impact of UI is driven more by symptom severity and psychological factors than by demographics. The most significant predictors were high leakage frequency (AOR = 4.25, *p* < 0.001), with women leaking several times daily being over four times more likely to experience severe social impact; higher perceived interference on a 0–10 scale (AOR = 1.28, *p* < 0.001); and experiencing anxiety or nervousness (AOR = 3.02, *p* = 0.005), which tripled the odds. Higher BMI was also a significant but weaker risk factor (AOR = 1.08, *p* = 0.002). Conversely, age, leakage volume, and incontinence during physical activity were not statistically significant predictors, suggesting their effects are mediated through the more direct pathways of frequency and psychological distress.

**TABLE 5:** Factors associated with a severe impact of urinary incontinence on social life among women in Saudi Arabia: a multiple logistic regression analysis.

Variable	Category	AOR	95% CI	*p* value
Age	(Continuous, per year)	1.02	0.98–1.06	0.301

BMI	(Continuous, per kg/m^2^)	1.08	1.03–1.13	0.002^∗^

Leakage frequency	≤ Once/week (Reference)	1	—	—
	Several times a day/always	4.25	1.95–9.27	< 0.001^∗^

Leakage volume	Small (Reference)	1	—	—
	Moderate/large amount	2.1	0.89–4.94	0.089

Interference score	(Continuous, 0–10 scale)	1.28	1.14–1.44	< 0.001^∗^

Physical activity leak	No (Reference)	1	—	—
	Yes	1.45	0.67–3.14	0.346

Anxious/nervous feeling	No (Reference)	1	—	—
	Yes	3.02	1.40–6.51	0.005^∗^

*Note:* Significant at *p* < 0.05 level. Statistically significant values were marked with an asterisk (^∗^) in the tables.

Abbreviations: AOR, Adjusted Odds Ratio; CI, Confidence Interval.

## 4. Discussion

UI, the involuntary leakage of urine, is a widespread health concern affecting up to 46.5% of women globally, particularly older adults [13]. In this study, “Frequent UI” specifically refers to leakage occurring ≥ 2–3 times per week, representing a clinically significant subgroup within the broader “Any UI” category. This condition has several risk factors, including aging, pregnancy, menopause, obesity, and chronic conditions such as hypertension and diabetes [14, 15]. Despite the emotional and social consequences of frequent UI, including shame, anxiety, and isolation, it is underdiagnosed and undertreated because of its stigma, particularly in Middle Eastern countries [16, 17]. On that basis, this study sought to evaluate the prevalence of UI among women in Saudi Arabia and its impact on their daily QoL.

Based on the systematic review of UI in Gulf countries [15], our overall prevalence of any UI (46.3%) is within a range of 20.3%–60.3%. Notably, the prevalence of frequent UI (27.5%) among our sample matches rates reported among women in Kuwait (26.4%) [18] and the United Arab Emirates (30.2%) [19], suggesting that a regional burden exists as well. These similarities most likely mirror similar sociodemographic factors such as high parity rates and increasing levels of obesity, as well as cultural influences that might affect both reporting and help‐seeking behaviors. In contrast, the overall prevalence of any UI in our analysis is lower than the 61.8% found among adult women in the USA [18]; this may relate to the younger age distribution of our sample population (66.6% less than 45 years) and exclusion of institutionalized elderly women, who have significantly higher rates of frequent UI. Age‐standardization is important for interpreting frequent UI prevalence across populations, as demonstrated in this comparison.

This study revealed a considerable burden of frequent UI, with 27.5% of participants experiencing frequent episodes. The pattern of leakage varied, with 64 (10.2%) of women leaking several times a day and 2.7% leaking all the time, indicating a cohort with severe symptoms. This prevalence is notably high, and a separate study focusing specifically on pregnant women in Saudi Arabia found an even higher overall UI rate of 31.3% [6]. Yet, it is substantially lower than the 61.8% overall prevalence found in the general US adult female population [18]. These contrasts likely reflect differences in sample age distribution, obstetric exposure, and methodological definitions, particularly because our study categorized only “frequent” (≥ 2–3 times weekly) cases, whereas others reported any frequent UI. So this stark difference is primarily due to distinct demographic differences across studies; the US data include older, nonpregnant women, where risk factors like advancing age and higher BMI, which were strongly associated with frequent UI, are more prevalent and cumulative. This finding is critical, as it moves beyond simple prevalence to quantify the profound daily burden of this condition. The fact that nearly one in eight incontinent women (12.9% of the affected cohort) experiences leakage this frequently underscores a significant clinical and quality‐of‐life challenge.

The current study identified several significant factors associated with frequent UI, particularly those related to chronic medical conditions and obstetric history. Participants with chronic diseases such as hypertension and diabetes were more likely to experience frequent UI, which may be attributed to neuropathic changes and reduced bladder control. A strong association was also noted with chronic constipation and chronic cough, both of which increase intra‐abdominal pressure and can lead to pelvic floor muscle strain over time, thus contributing to stress frequent UI [19]. In contrast, smoking status did not show a significant relationship with frequent UI in our sample, which may reflect differences in the prevalence of smoking among participants or variability in exposure duration. Reproductive factors demonstrated the strongest associations, as women with multiple pregnancies or vaginal deliveries showed increased frequent UI prevalence due to pelvic floor trauma and tissue stretching during childbirth. Cesarean delivery also contributed to frequent UI risk, likely because pregnancy itself weakens pelvic support structures. In addition, miscarriage, menopause, and family history were linked to higher frequent UI rates, reflecting hormonal influences and possible genetic predisposition. These findings are consistent with previous research, such as Zeas‐Puga et al., who similarly reported chronic diseases, age, vaginal and cesarean birth history, and parity as significant contributors to frequent UI [14]. Overall, our results emphasize that both pelvic floor stress and underlying health conditions play crucial roles in the development of frequent UI.

This study found that women’s QoL is significantly impacted by frequent UI. Physical activities were affected for over a quarter of respondents (27.7%), and social life was impacted in 25.4% of women. Psychological impacts were evident, with 7.2% experiencing depression and 21.0% feeling anxious. These factors form the fundamental causes of this anxiety and depression, which explains the broad and substantial effects of UI on day‐to‐day functioning and mental health. In line with this finding, Seshan V and Muliira reported that the QoL of women who reported UI was negatively affected, but the impact varied across dimensions [20]. Yağmur and Gül reported that the QoL among the women with frequent UI had been greatly affected. The most affected QoL domain was the social factor, with most feeling embarrassed to socialize [21]. These findings concur with ours, and therefore, it is acceptable to conclude that UI has a significant impact on the social, physical, and psychological well‐being of the affected women.

Our findings indicate that severe social impact from frequent UI is mainly driven by symptom severity and psychological distress, rather than demographic characteristics. Women with frequent leakage had much higher odds of severe social limitations (AOR = 4.25, *p* < 0.001). Those reporting anxiety or nervousness also faced increased risk (AOR = 3.02, *p* = 0.005). Each rise on the 0–10 interference scale—reflecting the participant’s perceived burden—significantly raised the likelihood of social impairment (AOR = 1.28, *p* < 0.001). These outcomes support existing evidence, including Minassian et al. [22], showing that bothersome and severe frequent UI symptoms are strongly linked to diminished QoL. Overall, both the physical burden of leakage and the psychological response are critical determinants of social functioning in affected women. This relationship aligns with established pelvic‐floor disorder theory: persistent leakage induces anticipatory anxiety, fear of public embarrassment, and social withdrawal [23]. These mechanisms worsen psychological distress and drive avoidance. This underscores that UI is both a physical and psychosocial disorder requiring integrated care.

A key novel contribution of this study is the identification of the factors that most strongly determine severe social impairment among women with frequent UI. While previous research has documented associations between UI and reduced QoL [10, 20, 21], our multivariable analysis reveals that severe social impact is driven primarily by symptom frequency (AOR = 4.25 for several times daily leakage), perceived interference (AOR = 1.28 per point on the 0–10 scale), and the presence of anxiety (AOR = 3.02). Importantly, demographic variables such as age and educational level did not remain significant after adjusting for these symptom‐related and psychological factors. This finding suggests that the social consequences of UI are mediated largely through the individual’s experience of symptom severity and emotional distress, rather than through demographic characteristics alone. To our knowledge, this is the first study in the Gulf region to quantify these independent pathways, providing a clear target for interventions: reducing leakage frequency and addressing anxiety may yield greater improvements in social participation than strategies focused solely on demographic risk factors.

These findings, from a public health perspective, are especially noteworthy for Saudi Arabia. Frequent UI is largely underreported due to social stigma, false beliefs about normal aging, and limited understanding of available treatments. This burden of care directly aligns with Vision 2030s objectives to improve women’s health, preventive care, and the QoL. Conducting routine screening for UI in primary care and gynecology clinics, reinforcing referral pathways to pelvic‐floor physiotherapy, and implementing culturally appropriate awareness campaigns may help to destigmatize frequent UI and significantly increase help‐seeking. Those steps are important for minimizing the clinical and psychological burden observed in this study.

Improving the QoL of women with UI in Saudi Arabia requires an integrated policy and service framework that addresses symptom burden, psychological distress, and social stigma together (Figure 4). The study’s findings that frequent leakage, higher perceived interference, and anxiety are the strongest determinants of severe social impact should be the core evidence guiding this framework.

**FIGURE 4:**
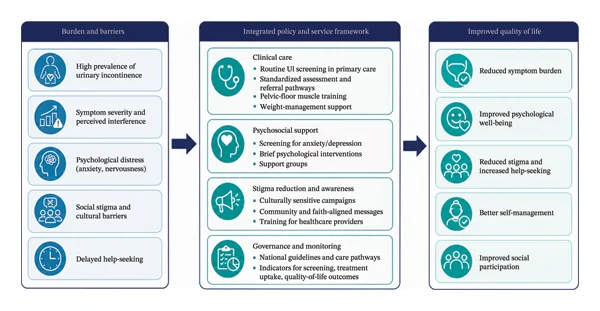
Improving the quality of life of women with urinary incontinence in Saudi Arabia through integrated policy and service framework.

### 4.1. Limitations

This cross‐sectional study had several limitations. First of all, the design provides only a single snapshot, making it difficult to draw causal conclusions and evaluate temporal patterns. Second, because participants may find it difficult to precisely recall previous instances of urine leakage, recall bias may compromise the reliability of the findings. Furthermore, the sample might not be representative of all females since the study used convenience sampling, which is prone to selection bias and limits generalizability. Finally, the identification of independent predictors of frequent UI and its effects on QoL may be jeopardized by unmeasured confounding variables. In addition, the mode of vaginal delivery (spontaneous vs. instrumental) was not differentiated in our questionnaire, precluding assessment of its distinct contribution to UI risk. Instrumental vaginal delivery (forceps‐ or vacuum‐assisted) is a well‐established risk factor for pelvic floor injury and subsequent UI (reference to be added). Future studies should specifically capture this variable to better elucidate its independent contribution to UI risk among women in Saudi Arabia.

While the ICIQ‐UI SF used in this study is a validated instrument, the newly developed sociodemographic and domain‐specific impact sections were not subjected to formal psychometric testing such as test–retest reliability or factor analysis, which represents a limitation of this study.

In addition, the use of an online survey may have introduced selection bias, as it primarily reached women with internet access and digital literacy, which likely contributed to the overrepresentation of university‐educated participants (77.9%) and may have underrepresented older or less educated women. Furthermore, despite quota stratification by region, the sample was disproportionately drawn from the Western and Southern regions, limiting the generalizability of findings to the entire Saudi female population. Future studies should consider mixed‐mode recruitment strategies, including face‐to‐face or telephone surveys, to achieve a more representative sample.

## 5. Conclusion

Frequent UI is highly prevalent among women in Saudi Arabia, seriously diminishing their social, physical, and psychological well‐being. The analysis highlights that the primary impact stems not only from physical symptoms but, more notably, from psychological distress and perceived burden. These findings underscore an urgent public health need to destigmatize frequent UI, increase awareness, and integrate accessible, culturally competent care to improve the QoL for affected women.

Psychological distress and perceived burden, rather than just physical symptoms, drive the most severe impacts of UI among women in Saudi Arabia. These results highlight an urgent public health need to address frequent UI by promoting destigmatization, raising awareness, and integrating accessible, culturally sensitive care to improve the QoL. These findings support a national framework that embeds routine UI screening in primary care, strengthens access to pelvic‐floor and weight‐management services, and integrates mental‐health support and culturally sensitive awareness campaigns to reduce stigma and improve the QoL.

**Nomenclature**
AOR: Adjusted odds ratio
BMI: Body mass index
CI: Confidence interval
ICIQ‐UI SF: International consultation on incontinence questionnaire–urinary incontinence short form
IQR: Interquartile range
MUI: Mixed urinary incontinence
QoL: Quality of life
SD: Standard deviation
SPSS: Statistical package for the social sciences
SUI: Stress urinary incontinence
UUI: Urge urinary incontinence
UI: Urinary incontinence

## Author Contributions

D.A. and E.M. conceived and designed the study, supervised data collection, and critically revised the manuscript for important intellectual content. A.H.A., A.F.H., L.A.A., and L.A. coordinated data collection, managed the database, and drafted sections of the Methods and Results. R.D.A., R.R.A., R.H.A., D.A.A., G.F.A., and D.M.A. contributed to questionnaire dissemination, data entry, and preliminary analysis. A.A.A., R.I.A., G.A.M.A, and O.S. provided clinical expertise in urology, contributed to interpretation of findings, and revised the manuscript for clinical relevance.

## Funding

This study was not funded.

## Disclosure

All authors reviewed and approved the final version of the manuscript and agreed to be accountable for all aspects of the work.

## Ethics Statement

Ethical approval was obtained from the Ethical Committee of the University of Bisha College of Medicine. Ref No: UB‐RELOC H‐06‐BH‐087/(03/04/46). All participants provided informed consent digitally before proceeding to the questionnaire. The consent form detailed the study’s purpose, voluntary nature, confidentiality assurances, and the right to withdraw. Anonymity and data security were maintained throughout the study.

## Consent

Please see Ethics Statement.

## Conflicts of Interest

The authors declare no conflicts of interest.

## Supporting Information

Additional supporting information can be found online in the Supporting Information section.

## Supporting information


**Supporting Information** STROBE Statement: This study was conducted and reported in accordance with the Strengthening the Reporting of Observational Studies in Epidemiology (STROBE) guidelines. A completed STROBE checklist is provided as a Supporting file (STROBE‐Appendix.pdf), detailing our adherence to each recommended item for cross‐sectional studies. The checklist maps the specific sections and page numbers in this manuscript where each STROBE reporting criterion is addressed, covering all the manuscript elements. Supporting File 1 (STROBE): STROBE checklist for cross‐sectional studies. This file contains the completed STROBE checklist, which maps each reporting criterion to the specific sections and page numbers in the manuscript where the criterion is addressed. The checklist demonstrates our adherence to the STROBE guidelines for observational research.

## Data Availability

The data used in this study are available from the authors.
