# Supplementary material for: Burden and Determinants of Urinary Incontinence and Its Quality‐of‐Life Impact Among Women in Saudi Arabia: A Cross‐Sectional Online Analysis
Source: Obstet Gynecol Int. 2026 Sep 30;2026:3195077. doi: 10.1155/ogi/3195077 (PMC13625042; doi:10.1155/ogi/3195077)
Supplement: Supplementary file 1 [file OGI-2026-3195077-s001.pdf]

STROBE Statement—checklist of items that were included in reports of the study

Section/Topic	Item No.	Recommendation	Page / Location in Manuscript
Title and abstract	1	(a) Indicate the study's design with a commonly used term in the title or the abstract.<br>(b) Provide in the abstract an informative and balanced summary of what was done and what was found.	Title page: "Prevalence, Risk Factors, and Quality-of-Life Impact…: A Cross-Sectional Study"; Abstract – Methods.<br>Abstract – all paragraphs (Background, Methods, Results, Conclusion).
Background/Rationale	2	Explain the scientific background and rationale for the investigation being reported.	Introduction – first 4–5 paragraphs (definition, global and Saudi prevalence, burden, under-reporting).
Objectives	3	State specific objectives, including any prespecified hypotheses.	Introduction – last paragraph stating aims to estimate prevalence/severity, identify risk factors, and assess QoL.
Study design	4	Present key elements of study design early in the paper.	Methods – "Study design" subsection (described as extensive cross-sectional analytical study).
Setting	5	Describe the setting, locations, and relevant dates, including periods of recruitment, exposure, follow-up, and data collection.	Methods – "Study design" and "Data collection procedures and instrument" (Saudi Arabia, 5 regions; May–Sept 2025).
Participants	6	(a) Give the eligibility criteria, and the sources and methods of selection of participants.<br>(b) Give the eligibility criteria, and the sources and methods of selection of participants.	Methods – "Sampling and sample size" (women ≥18 years, residents in Saudi Arabia; online recruitment with quotas).<br>Same as 6(a); no follow-up (cross-sectional).
Variables	7	Clearly define all outcomes, exposures, predictors, potential confounders, and effect modifiers.	Methods – "Operational definitions and measurements" and "Data Analysis" (UI, frequent UI, BMI, parity, menopause, etc).
Data sources/Measurement	8	For each variable of interest, give sources of data and details of methods of assessment (measurement).	Methods – "Data collection procedures and instrument" and "Operational definitions and measurements" (Arabic ICIQ-UI SF, self-report).
Bias	9	Describe any efforts to address potential sources of bias.	Methods – "Sampling and sample size" (quota stratification by region/age); "Data collection procedures…" (validated tool); Discussion – "Limitations".
Study size	10	Explain how the study size was arrived at.	Methods – "Sampling and sample size" (single-proportion formula, 50% prevalence, 95% CI, 5% error, final n=629).
Quantitative variables	11	Explain how quantitative variables were handled in the analyses.	Methods – "Operational definitions and measurements" and "Data Analysis" (BMI categories, UI frequency dichotomy, scales).
Statistical methods	12	(a) Describe all statistical methods, including those used to control for confounding.<br>(b) Describe any methods used to examine subgroups and interactions.<br>(c) Explain how missing data were addressed.<br>(d) If applicable, describe analytical methods taking account of sampling strategy.<br>(e) Describe any sensitivity analyses.	Methods – "Data Analysis" (descriptives; chi-square/Fisher; t/Mann–Whitney; multivariable logistic regression with confounders).<br>Not specifically reported; primary multivariable models for UI and severe social impact only.<br>Not explicitly described; denominators provided in tables (e.g., N=291 for leakage volume).<br>Methods – "Sampling and sample size" (quota sampling described); "Data Analysis" (standard logistic regression; no weights).<br>Not performed / not reported.
Participants	13	(a) Report numbers of individuals at each stage of the study…<br>(b) Give reasons for non-participation at each stage.<br>(c) Consider use of a flow diagram.	Methods – "Sampling and sample size" and Results opening: final analyzed sample n=629.<br>Not applicable/not reported for online survey.<br>Not included.
Descriptive data	14	(a) Give characteristics of study participants…<br>(b) Indicate number of participants with missing data for each variable of interest.	Results – Table 1 and text (age, nationality, BMI, region, marital status, education, occupation).<br>Results – Table 2 header (e.g., N=291) indicates missingness indirectly; not listed per variable.
Outcome data	15	Report numbers of outcome events or summary measures.	Results – Table 2, Figure 1, Figure 2 and text (prevalence of UI, frequency categories, interference, domain impacts).
Main results	16	(a) Give unadjusted estimates and, if applicable, confounder-adjusted estimates and their precision…<br>(b) Report category boundaries when	Results – Tables 3 and 4 (unadjusted % and p-values); Table 5 (AORs, 95% CIs for severe social impact).<br>Methods – "Operational definitions and

		continuous variables were categorized.<br><br>(c) If relevant, consider translating estimates of relative risk into absolute risk for a meaningful time period.	measurements" (age and BMI groups, UI frequency definition); tables' category labels.<br>Not applicable (prevalence in cross-sectional study).
Other analyses	17	Report other analyses done—e.g., analyses of subgroups and interactions, and sensitivity analyses.	Results – main multivariable model for severe social impact (Table 5); no additional subgroup/sensitivity analyses.
Key results	18	Summarise key results with reference to study objectives.	Discussion – first paragraphs (summary of prevalence, main risk factors, QoL impact vs objectives).
Limitations	19	Discuss limitations of the study, taking into account sources of potential bias or imprecision.	Discussion – "Limitations" subsection (cross-sectional design, selection and recall bias, unmeasured confounding).
Interpretation	20	Give a cautious overall interpretation of results considering objectives, limitations, multiplicity of analyses, etc.	Discussion – middle and final paragraphs (interpretation vs literature, mechanisms, and policy implications).
Generalisability	21	Discuss the generalisability (external validity) of the study results.	Discussion – "Limitations" and public-health implications (national coverage but online convenience sampling limits).
Funding	22	Give the source of funding and the role of the funders…	End matter – "Funding" (no external funding) and "Acknowledgments" (institutional support; no role in analysis/report).

Note: An Explanation and Elaboration article discusses each checklist item and gives methodological background and published examples of transparent reporting. The STROBE checklist is best used in conjunction with this article (freely available on the Web sites of PLoS Medicine at http://www.plosmedicine.org/, Annals of Internal Medicine at http://www.annals.org/, and Epidemiology at http://www.epidem.com/). Information on the STROBE Initiative is available at www.strobe-statement.org.
